# Cortical plasticity elicited by acoustically cued monetary losses: an ERP study

**DOI:** 10.1038/s41598-020-78211-7

**Published:** 2020-12-03

**Authors:** Aleksei Gorin, Elena Krugliakova, Vadim Nikulin, Aleksandra Kuznetsova, Victoria Moiseeva, Vasily Klucharev, Anna Shestakova

**Affiliations:** 1 International Laboratory of Social Neurobiology, Institute of Cognitive Neuroscience, National Research University Higher School of Economics, 20, Myasnitskaya St., Moscow, 101000 Russia; 2 Department of Neurology, Max Planck Institute for Human Cognitive and Brain Sciences, Leipzig, Germany

**Keywords:** Cognitive neuroscience, Decision, Perception

## Abstract

Both human and animal studies have demonstrated remarkable findings of experience-induced plasticity in the cortex. Here, we investigated whether the widely used monetary incentive delay (MID) task changes the neural processing of incentive cues that code expected monetary outcomes. We used a novel auditory version of the MID task, where participants responded to acoustic cues that coded expected monetary losses. To investigate task-induced brain plasticity, we presented incentive cues as deviants during passive oddball tasks before and after two sessions of the MID task. During the oddball task, we recorded the mismatch-related negativity (MMN) as an index of cortical plasticity. We found that two sessions of the MID task evoked a significant enhancement of MMN for incentive cues that predicted large monetary losses, specifically when monetary cue discrimination was essential for maximising monetary outcomes. The task-induced plasticity correlated with the learning-related neural activity recorded during the MID task. Thus, our results confirm that the processing of (loss)incentive auditory cues is dynamically modulated by previously learned monetary outcomes.

## Introduction

Why does a professional musician effortlessly discriminate the sounds of different instruments, while a random visitor to an opera house cannot? Why does the sound of our mother tongue immediately catch our attention in a foreign country? This and other examples of training-induced plasticity and adaptation to ecologically relevant stimuli are explained by the reorganisation of cortical representations in the human brain^1,2^. As shown by research with both animals^3^ and human^4–9^, training-induced plasticity, sometimes referred to as *directed cortical plasticity*^8^, may lead to the transient reorganisation of cortical maps through the enlargement of a representation area, often followed by the subsequent reversal of cortical expansion in parallel with the establishment of interconnections among specific cortical and subcortical areas^10^. In the current study, we investigated whether repeated behaviour associated with monetary outcomes also modulates perceptual processing. More specifically, we explored whether the monetary incentive delay (MID) task, which has been used extensively to investigate neural mechanisms of reward processing^11^, triggers task-induced cortical plasticity.

The MID task has been widely used to investigate reward processing in humans – approximately 200 functional magnetic resonance imaging studies have used the MID task to date^12,13^. The MID task requires an individual to respond quickly when a target stimulus appears after an incentive cue to obtain a reward or avoid losses. Importantly, the MID task allows for splitting reward processing into separate components: anticipation and feedback. The design of the task also allows for the manipulation of expected values and reward prediction errors. Using the MID task, numerous studies have demonstrated the role of the striatum, medial prefrontal cortex, posterior cingulate cortex and insula in the processing of incentive cues that indicate potential monetary rewards^11,13–16^. Surprisingly, the contemporary theoretical framework of decision-making largely overlooks experience-induced plastic changes in sensory areas. However, the modern theory of reinforcement learning seems to suggest that during the MID task, reward prediction error signals should drive the feedback-guided adaptive modification of behaviour^17^. Therefore, one can hypothesise that during the continuous MID task, participants could learn to better discriminate incentive cues associated with salient monetary outcomes due to rapid task-induced plasticity in the sensory cortex.

Important evidence of training-related neuropathic changes in auditory cortices was yielded by studies of mismatch negativity (MMN). MMN can be evoked by an oddball or a rare deviant auditory stimulus embedded in a sequence of a frequently presented standard stimulus^18,19^ or by using a roving standard paradigm in which a deviant sound becomes a standard one after some repetition, thus allowing as many stimuli as possible to be accommodated^20^. The ability to discriminate auditory stimuli is reflected in the time–amplitude characteristics of the MMN response. In addition to indexing sensory memory traces^19,21,22^, changes in the amplitude of MMN can also indicate the presence of long-term or permanent memory traces, for example, those for mother-tongue speech sounds^23–25^.

As it can be elicited irrespective of whether a participant is paying attention to the task or not^21,26^, MMN has become a widely used instrument in studies of various auditory functions: from automatic auditory discrimination to higher-order cognitive processes (e.g. language and speech)^27,28^, as well as training-induced plasticity^29–35^. Despite many studies where no attentional modulation of MMN^36–39^ was found, a number showed that the amplitude of MMN component can be modulated by attention under certain conditions^40,41^. For example, Woldorf et al.^41^, found that MMN amplitude was modulated by the channel-selective attention. This and other studies suggested that attentional effects take place when changes occur in the same feature in two auditory channels competing to be processes of the same MMN system^42^. Attention particularly affects MMN when the distracting task is of the same (i.e. auditory) modality^43^. In our study we distracted subjects from paying attention to the auditory stimuli by presenting video materials, thus dividing the channels of audio and video input information.

Although the idea that the sensory cortex directly participates in learning during classical conditioning is not novel in psychophysiology and well supported by experimental evidence^44^, relatively little is known regarding neural reorganisation in auditory cortical areas related to stimuli with different economic values. We assume that similar to plasticity during the learning of speech or music, neuroplastic changes could also take place when stimuli are being associated with economic values: for example, a slot machine sound could become associated with pay-out during a first visit to a casino. To test this hypothesis, we investigated whether the performance of the MID task changes the cortical representations of incentive (monetary) cues. For this, we investigated the relationship of task-induced cortical plasticity and a neural learning signal emitted during the MID task using another event-related potential (ERP) component called *feedback-related negativity* (FRN). Represented as a negative deflection of ERP with a fronto-central maximum occurring 240–340 ms after negative feedback, FRN is manifested during general mechanisms of performance monitoring that signal a reward prediction error^45–50^. Therefore, we hypothesised that individual differences in FRN recorded during the MID task could correlate with individual differences in the neural correlates of task-induced cortical plasticity.

To study MID-task-induced cortical plasticity, we further developed an auditory version of the MID task, in which sounds of different frequencies and intensities were used as incentive cues that signalled prospective monetary outcomes^51^. Different auditory cues indicated when a participant could lose 1 or 2 and 50 or 51 monetary units (MU). We also introduced different blocks of the MID task in which the perceptual learning of monetary cues was either relevant or not for maximising monetary outcomes. Auditory stimuli were composed of three pairs of incentive cues that predicted small and large losses: − 1 or − 2 MU (‘low losses’ context, *LL-trials*), − 50 or − 51 MU (‘high losses’ context, *HL-trials*), or − 1 or − 50 MU (‘widely varying losses’ context, *WL-*trials). Therefore, in the LL- and HL-trials, the difference between outcomes was small and irrelevant, whereas in the WL-trials, the participants were motivated to discriminate the cues for maximising monetary outcomes.

**Figure 1:**
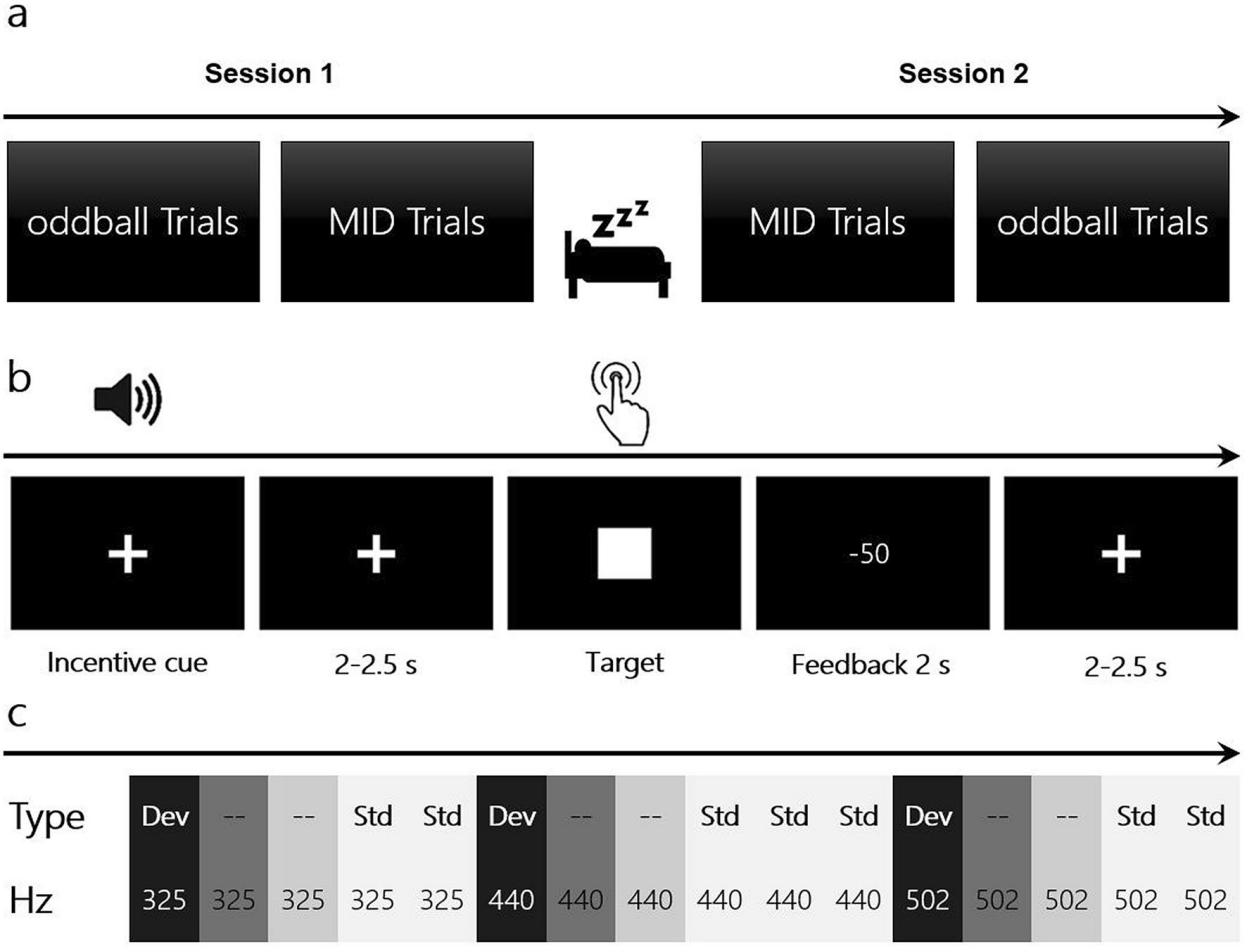
Schematic illustration of the experiment and its components. (**a**) Overall structure of the experiment. (**b**) Structure of a probe in the MID task. (**c**) An example of sound sequences in the roving oddball task.

The performance of the repeated MID task over the two consecutive days was framed using the oddball presentation of auditory cues in the MMN experiment (Fig. 1a). The combination of the MID and oddball tasks is referred to as a *session* throughout the text. Prior to each experimental day, a 5-min recording of a resting-state electroencephalogram (EEG) with eyes open was acquired from each participant. During the first session, the experiment started with the oddball task (approximately 30 min), followed by the MID task (approximately 45 min). During the second session, the MID task was followed by the oddball task. Therefore, we were able to study changes in the MMN component (oddball session 1 vs. oddball session 2) induced by the two-day MID task training.

We hypothesised that (1) the extensive MID task training would induce cortical plasticity, as indicated by the increased MMN in response to incentive cues that code salient monetary outcomes, (2) these plastic changes could be context-dependent and would take place only if learning is essential for maximising monetary outcomes and (3) plasticity-related changes in MMN might correlate with learning-related FRN recorded during the MID task.

## Results

### MMN-correlate of cortical plasticity (roving oddball task)

#### Sensor space

**Figure 2:**
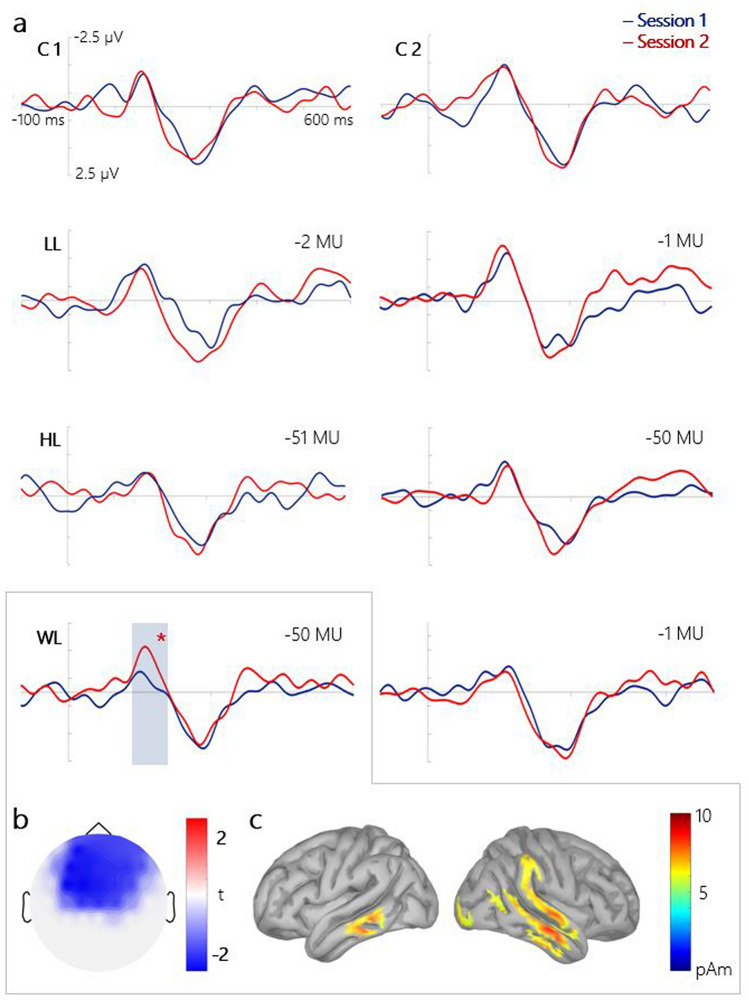
Evoked responses in the oddball task. (**a**) Grand-averaged difference waves (deviant *minus* standard) for the first and second sessions at the Cz electrode site. C1 and C2—high- and low-frequency control sounds. *LL* ‘low losses’ context, *LL-trials*; *HL* high losses’ context, *HL-trials*; *WL* widely varying losses’ context, *WL-*trials. (**b**) Cluster t-map for the dMMN (MMN in session 2 *minus* MMN in session 1) in response to large losses (− 50 MU) in WL-trials. (**c**) Source reconstruction of the dMMN in response to monetary cues that code large losses (− 50 MU) in WL-trials (186 ms post-stimulus).

We recorded EEGs before and after the two-day training in the MID task. Figure 2a shows the MMN responses obtained during the first and second sessions of the study. Notably, the MMN amplitude was enhanced only for the WL-trials (− 50 MU), i.e., only in trials where the participants could benefit most from learning to differentiate monetary cues that indicate two very different outcomes. This observation was supported by the significant interaction *Loss size* × *Day* × *Loss context* (F [2,56] = 3.17, *p* = 0.03)*.*

As was revealed in the post hoc comparisons, the MMN magnitude increased from − 0.56 µV in session 1 to − 1.6 µV in session 2, specifically in the WL-trials (Cz electrode, latency 170 ms). No significant differences between sessions 1 and 2 were observed for the MMN component evoked by the MID sounds or the control sounds (Fig. 2a). The observed that the task-induced enhancement of the MMN amplitude was bilateral, which was supported by the insignificant difference between the dMMN (MMN _session 2_ *minus* MMN _session 1_) at sites C3 and C4 (t = 0.4, p > 0.68).

To spatiotemporally localise the effects of the MID task on the MMN in response to the cue predicting large monetary losses (− 50 MU) in the WL-trials, we performed a paired cluster-corrected permutation test^52,53^. For the 140–200 ms time window, we found that the MMN amplitude significantly increased after the two sessions of the MID task (Fig. 2b). Thus, our results supported our hypothesis regarding induced plastic changes in the temporal cortex and demonstrated the context- and training-dependent increase in MMN in response to monetary cues that predict larger monetary losses.

#### Source analysis

We used source localisation analysis to further explore changes in the MMN evoked by monetary cues that predict large monetary losses (− 50 MU) in the WL-trials. At the latency of the maximal dMMN (186 ms, Fig. 2c), we observed right-lateralised activity at the temporal cortex, probably indicating task-induced neuroplastic changes in the auditory cortices. Next, we computed the mean current amplitude across the full set of cortical regions (the Desikan–Killiany atlas, as implemented in Brainstorm) and ranked them according to values of activation. The right middle temporal and bilateral inferior temporal gyri were most active during the oddball task (Table 1).

**Table 1:** Five cortical regions demonstrating the highest task-induced changes in MMN in response to incentive cues that code large (− 50 MU) losses in WL-trials of the MID task.

Structure	Hemisphere (R/L)	Average current, pA
Middle temporal cortex	R	3.17
Inferior temporal cortex	R	2.99
Superior temporal cortex	R	2.96
Temporal pole	R	2.61
Inferior temporal cortex	L	2.49

### FRN-correlate of learning in the MID task

**Figure 3:**
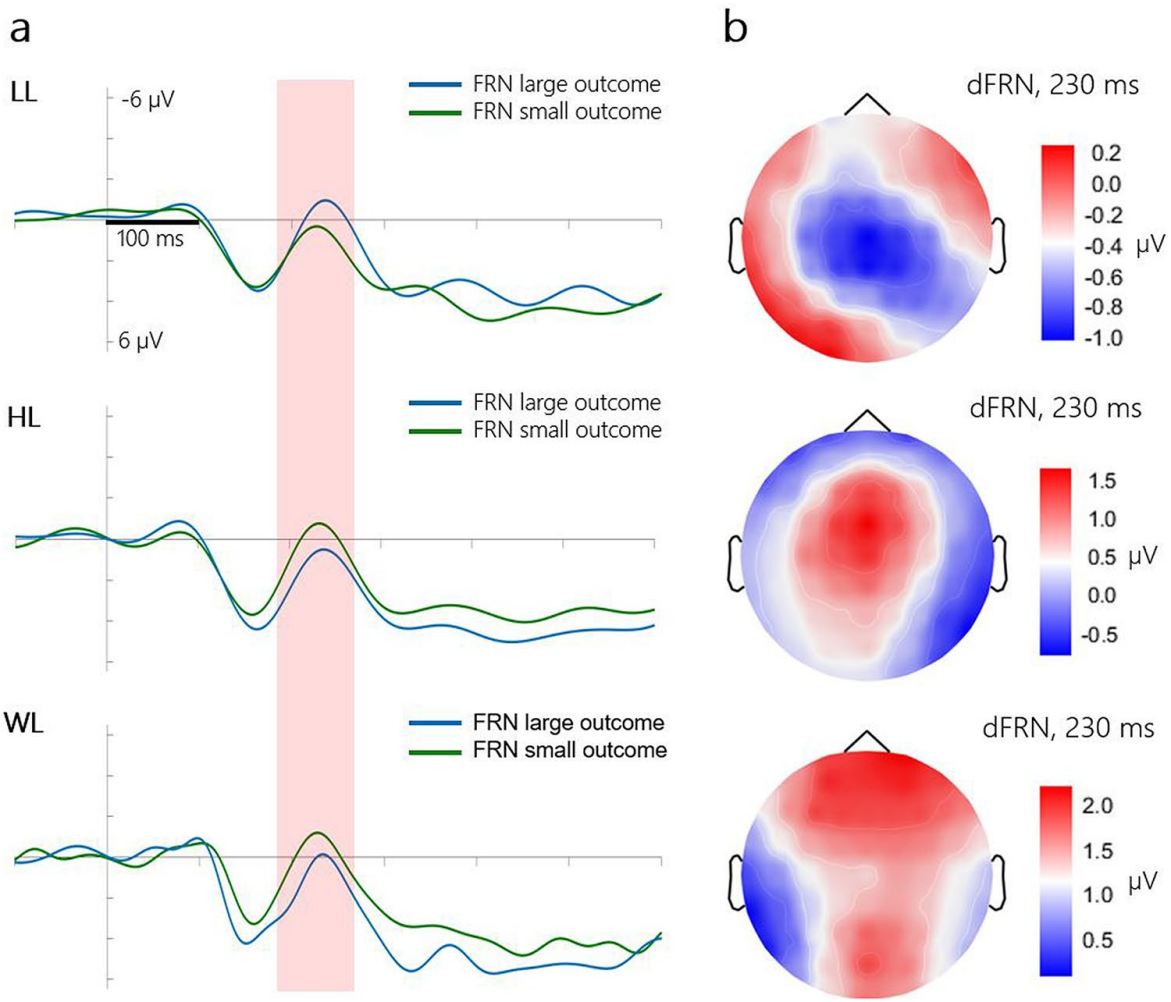
Evoked responses in the MID task. (**a**) Outcome-locked FRN waveforms at the Cz electrode site. Red rectangle indicates the time window of the FRN component latency. (**b**) Topography of dFRN (FRN _smaller outcomes_ *minus* FRN _larger outcomes_), 230 ms post-stimulus.

Figure 3a shows the FRN components (ERPs _positive outcomes_ *minus* ERPs _negative outcomes_) separately for the LL-, HL- and WL-trials. Notably, in the HL- and WL-trials, FRN was more negatively displaced in response to smaller losses than to larger losses, whereas in the LL-trials, the FRN pattern was reversed, as illustrated by the topographies (see Fig. 3b). A repeated measures ANOVA revealed the significant interaction *Loss size* × *Loss context*: F (2, 56) = 7.48, *p* = *0.0013*. Post hoc analysis confirmed that the FRN amplitude was more negative for smaller losses than for larger losses in the WL-trials (− 0.57 µV vs. 1.66 µV, *p* = 0.01). No other factors were significant.

The repeated measures ANOVA comparing the dFRN amplitudes (FRN _smaller outcomes_ *minus* FRN _larger outcomes_) revealed a significant effect of *Loss context.* Post hoc analysis showed that the dFRN was more negatively displaced in the LL-trials (− 0.99 µV) than in either the HH- (1.46 µV, *p* = 0.02) or DL-trials (2.24 µV, *p* = 0.002).

### MMN–FRN correlation

**Figure 4:**
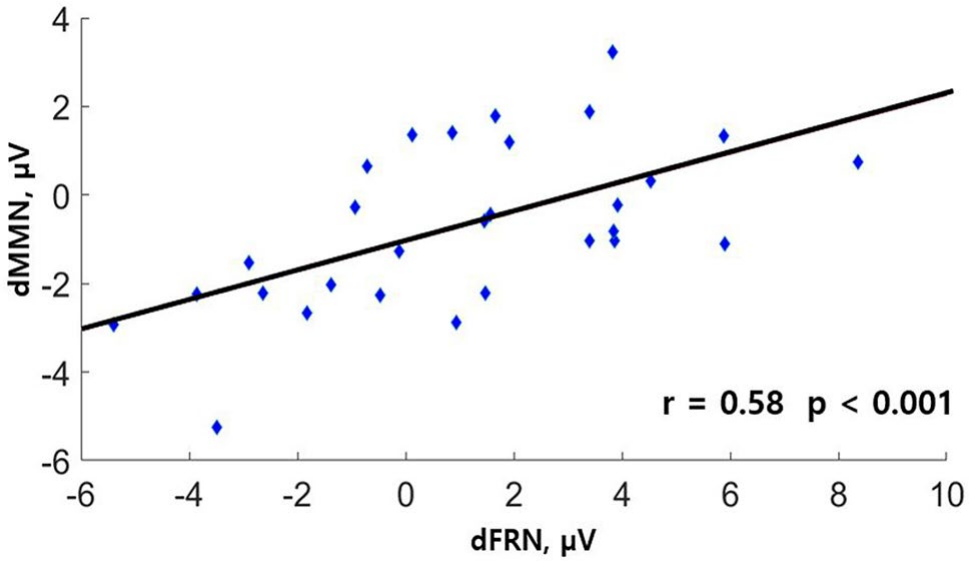
Correlation between the dMMN signature of cortical plasticity and the dFRN signature of reinforcement learning signals for the WL-trials.

We further checked whether individual differences in task-induced (neuroplastic) changes in MMN (oddball task) were associated with individual differences in the dFRN (MID task) as a neural signature of reward-based learning. Therefore, we correlated the dFRN amplitudes (200–260 ms) with the dMMN amplitudes (140–200 ms) in the WL-trials, where significant training-induced neuroplasticity was observed (Fig. 4). The Pearson correlation analysis revealed a significant positive association between dMMN and dFRN in the WL-trials (r = 0.56, *p* = 0.002). Additional analysis also revealed a significant correlation between dMMN and dFRN in the HL-trials (r = 0.42, *p* = 0.03) but not in the LL-trials (r = 0.22, *p* = 0.26).

### Behavioural results of MID task

At the behavioural level, we observed the facilitating effect of the session on subjects’s performance in the MID task. The mean RT shortened from 254 ms on the first day (session 1) to 238 ms on the second day (session 2)) (F [1, 28] = 13.72, *p* < *0.001*, η^2^_p_ = 0.33). The detailed results of the analysis could be found in the Supplementary materials.

## Discussion

The present study demonstrated that repeated exposure to monetary cues during the MID task results in training-induced cortical plasticity. We also found that the dMMN, an electrophysiological marker of this plasticity, correlated with the dFRN, a neural learning signal emitted during the MID task.

We compared the MMN responses with the auditory incentive cues before and after the two sessions of the MID task (separated by a night’s sleep), where the monetary cues indicated the payoff structure of the trial. From this, one can infer that the two sessions of the MID task resulted in altered preattentive discrimination of the incentive cues. Importantly, we observed significant training-induced changes in MMN only for the incentive cues, which were learned in the WL-trials, where large losses (− 50 MU) were intermixed with much smaller losses (− 1 MU). We found no training-related changes for the incentive cues, which were learned in the LL-trials (− 2 MU vs. − 1 MU) and in the HL-trials (− 51 MU vs. − 50 MU), where potential losses were very similar. Further, no cortical cortex plasticity was revealed under the control condition in which no ‘cue-outcome’ association was trained. Thus, we observed neural correlates of cortical plasticity only for the WL-trials, in which the participants could supposedly benefit most from learning to differentiate monetary cues that indicate two very different outcomes.

The observed context-dependent cortical plasticity can be explained by the behavioural saliency of large losses (− 50 MU) in the WL-trials. Strategically, it is only advantageous to correctly discriminate cues that predict larger losses from cues that predict smaller losses if the difference between the expected outcomes is significant. Logically, there is no motivation to carefully discriminate cues that predict almost the same outcomes (LL- and HL-trials). On the other hand, the WL-trials were the riskiest trials in our version of the MID task. Consequently, the participants were able to learn to discriminate incentive cues, particularly in the WL-trials, in order to ‘reduce’ risk because it is emotionally costly.

On the other hand, we did not observe changes in the WL trials that encoded a − 1 MU loss. In our task, the − 50 MU cue served as a predictor of a high loss, whereas the − 1 MU cue could be ignored since it weakly affected the total outcome. Nevertheless, we predict that such changes may appear in a paradigm that includes a direct association of each auditory cue with a specific behavioural response (i.e. when each monetary cue is associated with a unique response button). Plausibly, to enhance the effect on the MMN amplitude, precise cue-button association may be complemented with a second appearance of the auditory stimulus together with the feedback, or the sound may last throughout the trial. Also, longer and regular exposure to the MID task might boost cortical plasticity—auditory cues would elicit an enhanced response, while the sound would become behaviourally significant^54^.

Overall, our results suggest that the MID task might induce cortical plasticity changes in the sensory cortices, which might lead to better discrimination of monetary cues that indicate the prospect of large financial losses. Our interpretation of the results is supported by an earlier MMN study^31^ in which the participants who were able to detect a minimally deviant auditory pattern in a behavioural discrimination task demonstrated MMN in response to this deviant stimulus in a subsequent oddball task.

As suggested by theory, changes in the MMN amplitude induce the consolidation of memory traces and, therefore, functional cortical reorganisation^55^. Together with the aforementioned MMN findings of training-induced plasticity, the MMN results of our MID training fit a recently proposed systems-level theory of directed cortical plasticity^8^ in learning and memory, which could induce the transient enlargement of the representational areas during the learning of behaviourally salient sounds followed by task-specific maintenance. In paradigms where sounds are used as conditioning stimuli, the training results in associative representational plasticity, which could facilitate responses to the cue stimuli^56^. According to the representational plasticity theory, the tuning of the neurons in the auditory cortex may shift towards the parameters of the conditioned stimulus, thus biasing the sensory system to distinguish the behaviourally important stimulus^6,9,57,58^. Importantly, auditory plasticity can occur in the absence of any enlargement of a representation area; therefore, an observed cortical magnification in representation due to a conditioned stimulus may be transient and may not be necessary to support learning^7,8^.

Previous studies have used the MMN component to show plastic changes in the auditory cortices, predominantly for sociobiologically relevant sounds, such as speech- and language-specific sounds^19,59–68^. For example, a seminal magnetoencephalographic study showed that Finns produce a larger MMN amplitude for a Finnish phoneme than for a non-native (Estonian) phoneme^16^. Other MMN studies have reported superior auditory processing in musicians as compared with non-musicians^35,38–45^. For example, expert jazz musicians had a larger and earlier magnetic counterpart of MMN (MMNm) response to subtle deviations in rhythm than musically inept participants^69^. Even mere daily exposure to the phone ringing elicits an enhanced oscillatory response^54^.

To the best of our knowledge, our study is the first to report auditory cortical plasticity for incentive cues associated with the anticipation and delivery of monetary outcomes. Our results indicate that performance in the MID task, which is often used to investigate neural mechanisms of decision-making and reward processing, could evoke cortical neuroplasticity manifested in the enhanced MMN.

Popular neurobiological models of decision-making often assume that sensory inputs to decision-making neural networks are stationary. In this respect, it is important not to underestimate the impact of plastic changes in sensory cortices evoked by repeated decisions. To promote survival, the brain learns perceptual stimuli that signal potential rewards^70,71^. Many visual studies have shown that reward facilitates voluntary attention to task-relevant stimuli^72–77^. Other studies have demonstrated a processing advantage for stimuli associated with rewards: both for positive and negative outcomes^58–60^. For example, acquired emotional meaning (often as a result of reinforcement learning) is associated with shorter RTs and lower error rates^78,79^. Overall, reward learning modifies the attentional priority of stimuli and allows them to compete for selection even when they are not salient and task-irrelevant^80^. In fact, neural activity related to reward expectancy has been observed in sensory cortices^70,76,81,82^. For example, event-related magnetic fields in the visual cortex have been shown to code the expected reward value 155 ms after the cue, indicating that reward value modulates early sensory processing^83^. Furthermore, monetary value modulates the neural response to an object in visual areas of the brain, even when attention is diverted^84^. In this regard, more studies of reward-associated plasticity across different sensory functions would be extremely valuable for clarifying the neural mechanisms of the interplay of decision-making and attention during the learning of incentive cues.

In our study, the source modelling of the MID-task-induced dMMN showed activation in the temporal cortex that has been proposed as one of the MMN’s generators^85–88^: the right middle temporal and bilateral inferior temporal gyri were most active during the oddball task. This finding of right-hemisphere activations of the middle and inferior temporal gyri is in line with previous studies of pitch processing^89^. A left-hemisphere MMN predominance was observed by Kujala et al. (2003) for deviant Morse patterns as a result of intensive Morse-code training. Some studies have suggested that laterality of MMN can be explained by the stimulus-feature association. In linguistic contexts (grammatical or phonological), left-lateralised networks are activated^27,90^, whereas in musical contexts, right lateralisation is observed^89^. However, when musical sounds acquire meaning, the responses to musical stimuli transfer to the left hemisphere. In the study by Vuust (2005), the musicians’ MMNm for these deviations was left-lateralised, whereas the (smaller) MMNm of the non-musicians was right-lateralised. The authors suggested that this left-lateralisation reflects the functional adaptation of their brain to a task of communication which, in these musicians, is much like that of language when they play jazz together, with subtle rhythmic deviations forming signals of musical communication for them. In our study, the training and learning of stimuli could perhaps lead to a meaningful categorisation of which ones activated the semantic network.

The EEG-based source analysis of our study does not render very high precision. Therefore, the contribution of the frontal sources of MMN, which are more related to an involuntary attention switch caused by auditory change^21,26,85,91–93^, could not be completely ruled out in our experiment. Further studies employing functional magnetic resonance imaging (integrated with EEGs) or magnetoencephalography will be necessary to further clarify all the sources of training-induced activity.

To detect training-induced changes, we recorded MMN on two separate days as sleep plays a consolidating role in forming memory representations^94,95^. According to the interference hypothesis, sleep facilitates learning because it prevents interference from ongoing sensory input^95^. Sleep causes an additional increase in the amplitude of MMN after the sound discrimination training, thus facilitating the slow neural plastic changes that underlie preattentive sound processing^96^. However, previous ERP studies have shown that plasticity can develop very rapidly^27,28,31,35^. For example, in the study by Gottselig and colleagues^31^, it took less than 6 min to observe the enhancement of the MMN. On the other hand, Alain and co-authors published MEG evidence that changes in auditory evoked fields are more salient after sleep, compared to same-day recordings^97^.

We also found a significant correlation between the individual differences in neural markers of training-induced changes (dMMN) and the individual differences in the dFRN marker for learning during the MID task. This positive correlation was observed only in the WL-trials: the larger dFRN recorded during the MID task correlated with larger training-induced plastic changes, as indicated by the dMMN in response to incentive cues during the oddball task. This correlation further supports our observation of a selectively induced plasticity in the auditory cortex that is driven by the MID task. This corroborates previous studies that have robustly demonstrated that FRN amplitude predicts the effectiveness of the learning obtained in various probabilistic learning studies^50,98–102^. Thus, our findings provide evidence of an association between training-induced cortical plasticity and neural performance monitoring mechanisms at the individual level.

There are different theories regarding the neural mechanism underlying FRN. For example, FRN may respond to unexpectedness, i.e., reflecting a general signal of expectation violation irrespective of valence^103^. It has also been proposed that the generation of FRN is linked to phasic (reinforcement learning) signals produced by the dopamine system and projected to the dorsal anterior cingulate cortex^45,104,105^. This hypothesis is supported by the neural activity of the dopaminergic system, which responds to situations that are better or worse than expected^46,106^. Notably, the FRN pattern in response to losses differs from that in response to the omission of gains. In the case of gains, the omission leads to a more negative deflection^107,108^, whereas loss avoidance leads to a more negative deflection. A recent study also demonstrated that FRN in response to the omission of outcomes was larger in the gain blocks than in the loss blocks, reflecting the context dependence of FRN^109^. Our results show that the dFRN as a difference between FRN in response to smaller and larger losses varied in different trial types: the dFRN was negative in the LL-trials and positive in the HL- and WL-trials. Our results indicate that in the context of losses, FRN is modulated by reference points and expectations.

In our recent study^110^ that used an auditory MID task with gain outcomes, no training-induced effect on MMN was found. To find neuroplastic changes in the auditory cortex, we substantially modified the MID task: we introduced different trial types and used losses instead of gains. In this way, we enhanced the behavioural salience of the monetary cues, particularly in the WL-trials, where better discrimination of cues (− 50 MU vs. − 1 MU) was more behaviourally relevant. Importantly, expectations of rewards and losses activate differently the neural circuitry involved in reward anticipation^13,111^. Although this question is widely debated^112,113^, there is evidence in favour of the division of human learning systems into reward and punishment modules^114^. Thus, further studies are needed to extend our results to the gain domain.

Overall, we showed that the monetary cues that indicate salient financial losses during the auditory version of the MID task trigger plastic changes in the temporal cortex. These experience-induced plastic changes were indicated by the enhanced MMN response recorded during a passive oddball paradigm following two sessions of the MID task. Our results indicate better preattentive discrimination of cues that code the prospect of salient financial losses. To the best of our knowledge, this is the first neurophysiological evidence of enhanced preattentive auditory discrimination of monetary cues that code salient financial outcomes. We also found a correlation of the MMN marker for experience-induced cortical plasticity with the FRN marker for reinforcement learning: the larger learning-related FRN during the MID task predicted the stronger training-induced MMN after the second day of training. In light of our findings, it is not surprising that slot machines today feature about 400 sound effects on average, which are carefully constructed^115^. Thus, the results of the present study further contribute to our knowledge of the important role of sensory cortices in value-based decision making.

### Study limitations

There are a number of limitations to this study that could be worthy of considering for the future progress of studies of task-induced neuroplasticity in the context of economic theory. In the present study, we found that two sessions of the auditory MID task evoked a significant enhancement of MMN in response to monetary cues predicting large monetary losses only in the context of small losses, specifically, when monetary cue discrimination was essential for maximising monetary outcomes. As revealed by the combination of the oddball and MID tasks, reinforcement learning mechanisms might sharpen the representation of task-relevant stimuli and attention allocation. However, the MID task is an instrumental-reward task, which requires participants to accomplish an ‘instrumental’ action correctly in order to obtain the reward^11,116,117^. This instrumental action is linked to a single expected value, in contrast to decision-making tasks that require participants to actively select between more than one action, with different expected values on any given trial. Furthermore, during the MID task, participants do not make mistakes, and reinforcement learning could only be monitored indirectly through reaction times. Information regarding correct and incorrect choices is essential for the modelling of reward-based learning. Therefore, in the MID task, information about individual learning is very limited.

In this study, we used sound pitches composed of three frequencies, following our initial experiments^51,110^. On the other hand, in animal study^118^ of task-induced auditory cortex plasticity to multitonal chord stimuli, experimental results suggested that the observed plasticity might be explained as a superposition of changes induced by each individual tonal component composing the chord.Therefore, plasticity evoked by the harmonics of a lower sound could be in conflict with changes induced by the central frequency of a higher sound. This could, hypothetically, affect the results of MID and oddball tasks.

In our study, we used a two-day paradigm, allowing our participants to sleep before we tested for the effects of MMN. This approach may be supported by the findings of Alain and colleagues^97^, where the most pronounced changes in event-related fields were observed on the next day. However, investigating the rapid plastic changes of evoked responses such as those in Alain and colleagues^28^ or Kluge and colleagues^119^ is equally important in the light of the contemporary theory of instrumental task-induced neuroplasticity^9^.

Finally, in this study, we used a common head model to estimate the source of the MMN. This approach allowed us to assume that the temporal cortex was a probable site of the plasticity. However, for a detailed study of MMN-related plasticity in a source space that allows for drawing firm conclusions, a more spatially precise method, such as MEG with individual brain models, is required. Using a fine instrumental approach would allow to resolve another important issue associated with the effect of attention on neural plasticity associated with the MID. Contribution of the frontal sources of MMN, which are more related to an ‘involuntary attention switch’ caused by auditory changes, could not be completely ruled out in our study. To perform a better control of attention allocation during the oddball task would be absolutely necessary to rule out any possibility of contaminating our findings by the effect of attention modulation.

### Future research directions

To further investigate plastic changes in MMN amplitude, we suggest that future studies should take into account the aforementioned limitations. To study the behavioural correlates of ERP changes, the adaptive target time algorithm should be replaced by a different solution to encode the outcome probability. One possible direction could be to adapt a variant of a lottery paradigm, e.g. a variation of the two-armed bandit task involving the active participants’ choices (Behrens et al., 2007) to auditory modality and perform a concurrent MEG-EEG study where participants will be offered to choose between two options with different expected values encoded in sound cues. This approach will allow one to study the dynamics of the behavioral aspect of reinforcement learning and evaluate individual learning parameters through the mathematical modeling techniques. Brain stimulation methods, on the other hand, could be helpful to understand the causal relationship between dFRN and dMMN signals. A follow-up study would also be well advised to investigate the role of attention in task-induced changes in MMN, for example using a modified dichotic MMN paradigm^40,41^.

## Methods

### Participants

Twenty-nine healthy, right-handed participants (12 males, mean age 23) participated in this study. All of them had normal or corrected-to-normal eyesight. Prior to the experiment, each participant signed an informed consent form. This experiment was approved by the local ethics committee.

### Auditory stimuli

We used eight harmonic sounds composed of three sinusoidal partials as stimuli. The fundamental frequencies of the sounds were as follows: 272, 325, 381, 440, 502, 568, 637 and 711 Hz (equal to 370, 430, 490, 550, 610, 670, 730 and 790 mel, respectively; therefore, minimal step between the sounds was equal to 60 mel). The frequency of the second and third partials were two and three times higher, respectively, than the fundamental frequency (e.g. 381, 762 and 1143 Hz). Compared with the first partial, the intensities of the second and third partials were reduced by 3 and 6 dB, respectively. The sounds were 200 ms long, including 5 ms rise and fall times, and had a 70 dB intensity controlled by a sonic level meter. Stimuli were generated with the PRAAT software.

### MMN recording: roving oddball task

We used the roving oddball paradigm^15,100^ to record training-induced changes in the MMN component. Participants were instructed to read a book of their own choosing during the experiment and ignore the auditory stimuli. During the roving oddball task, sounds were presented binaurally with earphones at a constant intensity level of 70 dB. Across the trials, eight tones (sounds) formed a roving oddball sequence with 4–6 tone repetitions at each possible carrier frequency, where the first occurrences of a given frequency were considered deviants, while the rest (except for the second and third) were considered standards (Fig. 1c). The interstimulus interval varied from 600 to 800 ms. Each sound type was presented randomly 50 times under the constraint that each consecutive sound was required to be from 1 to 3 steps higher or lower than the previous (two steps or 120 mel on average). It should be noted that the lowest and highest sounds were not used in the MID task and were analysed separately as control condition stimuli that had no association with monetary outcome. The task lasted approximately 30 min.

### Auditory MID task

During the MID task (Fig. 1), the participants were instructed to press the button (which was the same for all conditions) as fast as possible when a target (a white rectangle) appeared at the centre of the screen. After a delay, they received feedback: if the trial was successful, a green ‘0’ appeared on the screen; otherwise, the program informed the participants about the sum of money they had lost (e.g. − 50 MU). The monetary outcome was encoded by an auditory cue that preceded the appearance of the target by 2000–2500 ms. The trial was judged as successful if the button had been pressed before the target disappeared. Outcome probability was manipulated by adjusting the duration of the target stimulus through an adaptive timing algorithm that followed the participants’ performance such that for each trial type, they would succeed on 60% of the trials in each trial type.

In order to discover the changes in cortical responses associated with expected value, we framed losses in three different contexts: low, high and widely varying losses (LL, HL, WL), where one could lose 1 or 2, 50 or 51, and 1 or 50 MU, respectively. The task was split into six blocks: two blocks per context. In each block, only two stimuli and two corresponding monetary outcomes were used (see Supplementary Fig. S1).

If a participant pressed a button multiple times or pressed the button prior to the appearance of the target, the feedback screen returned ‘!!!’ in red, which indicated a performance error. This trial was then not counted as finished and was rerun during the game to maintain the 50 trials per cue proportion.

Prior to the MID session, participants received an endowment of 4000 MU (~ 70 USD). They were instructed that they might lose a part of the initial endowment during the game and that they could complement their compensation for participating in the experiment with any remaining balance.

Importantly, six auditory stimuli (325, 381, 440, 502, 568 and 637 Hz) composed three pairs of incentive cues that predicted small and large losses: − 1 or − 2 MU (‘low losses’ context, *LL-trials*), − 50 or − 51 MU (‘high losses’ context, *HL-trials*), − 1 or − 50 MU (‘widely varying losses’ context, *WL-trials*). Therefore, in the LL- and HL-trials, the difference between the outcomes was equal to 1 MU, which was irrelevant in the context of the (4000 MU) initial endowment, whereas in the WL-trials, the difference between the outcomes was equal to 49 MU; therefore, the participants were more motivated to discriminate the cues, i.e., perceptual learning was only relevant for maximising monetary outcomes in the WL-trials. This design allowed us to separate the effect of context (behavioural relevance) on participants’ behaviour from the effect of outcome size. Each pair of auditory monetary cues was randomly presented within mini-blocks of 50 trials: LL-trial blocks, HL-trial blocks or WL-trial blocks. Overall, each MID task session consisted of six mini-blocks such that each of the three context types (LL-, HL- and WL-trials) appeared twice during the experiment.

Two additional sounds presented in the oddball task (272, 711 Hz) served as control stimuli and were not used in the MID task (Table 2). The sample size was too small for full randomisation; therefore, we counterbalanced (acoustic) incentive cue-outcome mapping using six basic combinations to exclude the confounding effect of pitch on the MMN. Thus, the cue-outcome mapping between the lower/higher frequencies and the lower/higher outcome magnitudes was counterbalanced across participants (for details, see Supplementary Table S1).

**Table 2:** An example of the incentive cue-outcome mapping during the MID task.

Incentive cue (frequency, Hz)	Outcome magnitude (MU)	Trial types
325	− 1	LL-trials
381	− 2	
440	− 50	WL-trials
502	− 1	
568	− 51	HL-trials
637	− 50	
272	Control condition—these stimuli were not used in MID task	
711		

*LL-context *Low losses,* HL-context *high losses, *WL-context *widely varying losses*.*

Incentive cue-outcome associations were counterbalanced across participants (for details, see Supplementary Materials, Table S1).

The duration of the first target stimulus in the main experiment was based on the mean reaction time in a short pre-test prior to the MID session, where the participants quickly responded to the same target that appeared at the centre of the screen for 400 ms. Each MID task was preceded by the training part. During the training, the participants learnt to associate the auditory cues they were exposed to with the monetary outcomes. On the screen, the participants were exposed to two images indicating possible monetary losses. After the auditory signal, they picked the corresponding sum using one of two buttons (left and right arrows) and received feedback (Supplementary Fig. S2). If the participant was successful in the last 8 out of 10 trials, the training stopped, and the main MID task started.

### Analysis of behavioural data

To test the behavioural changes evoked by two sessions of the MID task that implemented different loss contexts, we used a 2 × 2 × 3 repeated measures ANOVA with the following factors: *Loss size* (large vs. small), *Day* (session 1 vs. session 2) and *Loss context* (*LL-, HL- and WL-trials*).

### EEG acquisition and preprocessing

We continuously recorded EEGs during all sessions—oddball and MID tasks—of the experiment with the BrainProducts ActiChamp system, using 60 active electrodes positioned according to the extended version of the 10–20 system. Channels were referenced against the averaged signal from two mastoid electrodes. Electrooculograms (EOGs) were recorded using electrodes placed below the right eye and on the left outer canthi. We applied an online 50 Hz notch filter; the ground electrode was placed on the Fpz site. The electrode impedance was kept below 5 kΩ. We performed EEG analysis using the Brainstorm toolbox^120^. Raw recordings were visually inspected for artefacts. Noisy segments were excluded from further analysis. The EEG data were filtered between 1 and 40 Hz. To correct for eye-movement artefacts, we used JADE independent components analysis (ICA). The eye-movement components were removed according to their topography and correlation with the EOG. After preprocessing, we imported − 200 to 800 ms baseline-corrected (− 100 to 0 ms) epochs locked to the sound onset.

### Statistical analysis of the MMN results in the roving oddball task

To identify and measure MMN, we subtracted the ERPs of standard sounds from the ERPs of deviant sounds. To detect the effect of training-induced plasticity on auditory discrimination, we performed a 2 × 2 × 3 repeated measures ANOVA with the following factors: *Loss size* (large vs. small), *Day* (session 1 vs. session 2) and *Loss context* (*LL-, HL- and WL-trials*). The ANOVA was performed on the mean difference wave’s amplitudes at the Cz electrode site using a 170 ± 30 ms time window that corresponds to the MMN peak on the grand-averaged ERP. Post hoc tests were performed using the LSD test. To spatiotemporally localise the effects of *Loss size, Day* and *Loss context* of the training-induced plasticity on MMN, we performed a paired cluster-corrected permutation test^53^ implemented in the Brainstorm software: the cluster inclusion threshold was set to *p* < 0.05 with 1000 permutations over the full epoch time windows. The cluster *p*-values were defined separately for the positive and negative clusters as the probability of observing a cluster with a higher mass. To localise the effects of *Loss size, Day* and *Loss context* in the source space, we used a weighted MNE method^121^ implemented in the Brainstorm software, and projected the grand-averaged difference in MMN activity onto a standard head model (6000 vertices) using a standard electrode layout. To test the a.m. main effects in the source space, we used the 140–200 ms time window defined in the sensor-space analysis.

### Statistical analysis of the FRN results in the MID task

To extract the FRN component, we subtracted the ERPs of ‘no loss’ (positive, [+ 0 MU] – zero-value outcomes) outcomes from the ERPs of ‘loss’ (negative) outcomes collected in two sessions: (FRN = ERPs _positive outcomes_ *minus* ERPs _negative outcomes_). The individual FRN amplitudes were computed for the Cz electrode site as a mean difference wave’s amplitude within the 230 ± 30 ms time window, which corresponds to the FRN peak latency. For each participant, we also calculated the individual dFRN by comparing the FRNs to the small and large losses (dFRN = FRN _larger loss_ *minus* FRN _smaller loss_). We analysed the dFRN amplitudes using a three-way repeated measures ANOVA with the factor *Loss context* (*LL-, HL-* and *WL-trials*). All ANOVA tests were performed using StatSoft STATISTICA 12 software.

### Correlation of FRN and MMN

To study the relationship between reinforcement learning and training-induced cortical plasticity, we correlated individual dFRN (WL-trials) with individual changes in MMN (− 50 MU, WL-trials) (dMMN = MMN _session 1_ *minus* MMN _session 2_) at the Cz electrode site using Pearson correlation analysis.

### Ethical approval

The study was approved by local ethics committee of National Research University Higher School of Economics and was performed according to relevant regulations. All participants read and signed an informed consent prior to the experiment, and received a monetary reward as compensation for participation.

### Informed consent

All participants were familiarised with the experimental procedure and signed the informed consent form**.**

## Supplementary information

Supplementary Information

## Data Availability

The datasets analysed during the current study are available from the corresponding author on reasonable request.
