# Supplementary material for: Cortical plasticity elicited by acoustically cued monetary losses: an ERP study
Source: Sci Rep. 2020 Dec 3;10:21161. doi: 10.1038/s41598-020-78211-7 (PMC7713235; doi:10.1038/s41598-020-78211-7)
Supplement: Supplementary file 1 — Supplementary Information [file 41598_2020_78211_MOESM1_ESM.docx]

**Supplementary materials**

**Cortical plasticity elicited by acoustically cued monetary losses: an ERP study**

**Alexey Gorin1, Elena Krugliakova^1^, Vadim Nikulin^1,2^, Aleksandra Kuznetsova^1^, Victoria Moiseeva^1^, Vasily Klucharev1 & Anna Shestakova1**

^1^ International Laboratory of Social Neurobiology, Institute of Cognitive Neuroscience, National Research University Higher School of Economics, Russian Federation

^2^ Department of Neurology, Max Planck Institute for Human Cognitive and Brain Sciences, Leipzig, Germany

1. **Supplementary methods and descriptive statistics**
2. **Supplementary results**
3. **Supplementary methods and descriptive statistics**

**Auditory MID task**


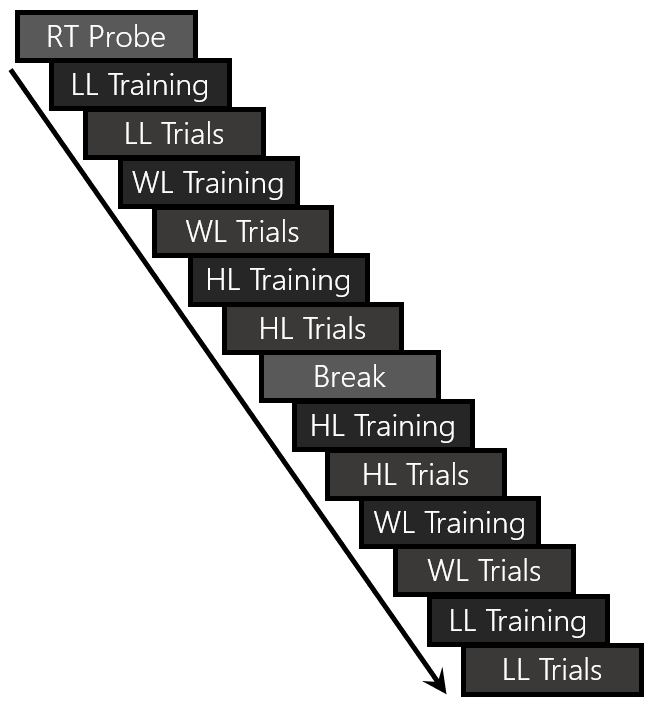


**Figure S1**. A sample trial sequence for a session of the MID task. The MID task was preceded by the reaction time (RT) test, which determined the duration of the first target stimulus in the main experiment. The MID task was split in two parts separated by a 5-min break. Each part consisted of three blocks of different trial types: WL-trials, LL-trials, HL-trials, which corresponded to low losses, high losses, or widely varying losses contexts. The order of blocks was counterbalanced across participants. Each block was preceded by training trials.


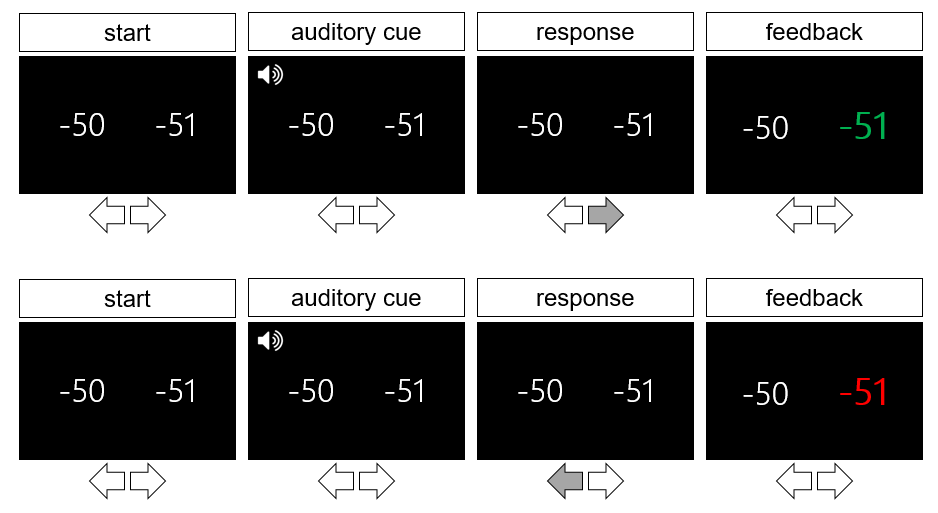


**Figure S2**. Sample trial screens for the training. During the training, participants learned to associate auditory cues with specific monetary outcomes. On the screen, the participants were exposed to two images indicating possible monetary losses. After the auditory signal (cue), they picked the corresponding sum using one of two buttons (left and right arrows) and received a feedback. Positive feedback was shown in green and negative in red. If the participant was successful in the last eight out of ten trials, the training stopped and the main MID task started. Grey colour of the arrow indicates a hypothetical response of a participant.

**Table S1**. Examples of cue-outcome mapping. To exclude a confounding effect of pitch on the MMN, we counterbalanced acoustic cue-outcome associations using six combinations.

| **Frequency, Hz** | **Values, MU** | | | | | |
| --- | --- | --- | --- | --- | --- | --- |
| Сue-outcome mapping | 1 | 2 | 3 | 4 | 5 | 6 |
| 325 | −1 | −51 | −50 | −1 | −51 | −50 |
| 381 | −2 | −50 | −1 | −2 | −50 | −1 |
| 440 | −50 | −1 | −51 | −51 | −50 | −1 |
| 502 | −1 | −2 | −50 | −50 | −1 | −2 |
| 568 | −51 | −50 | −1 | −50 | −1 | −51 |
| 637 | −50 | −1 | −2 | −1 | −2 | −50 |

1. **Supplementary results**

***Participants’ payoff***

The maximal payoff in Session 1 was 1661 MU. Thus, during the MID, task participants could lose at most 2339 MU from their initial endowment (4000 MU _initial endowment_ – 1661 MU _payoff_). The maximal payoff in Session 2 was 1455 MU. Thus, during the MID task, participants could lose at most 2545 MU from their initial endowment (4000 MU _initial endowment_ – 1455 MU _payoff_).

***Behavioural results of MID task***

The repeated measures ANOVA revealed a significant effect of the factor *Day* on the mean reaction time (RT) (F [1, 28] = 13.72, *p < 0.001*, η^2^_p_ = 0.33). The mean RT decreased from 254 ms on the first day (session 1) to 238 ms on the second day (session 2). Therefore, we observed a significant training effect during the MID task. The factor *Loss size* also modulated RTs: RT was significantly shorter in trials with larger expected losses than in trials with smaller expected losses (245 vs. 247 ms; F [1, 28] = 6.56, *p = 0.016*, η^2^_p_ = 0.19). We also found the significant interaction *Loss size × Day × Loss context* (F [2, 56] = 4.36, *p = 0.02*, η^2^_p_ = 0.14). The least significant difference (LSD) post hoc comparisons revealed that during session 1, RT was shorter in trials with large losses (−50 MU) than in those with small losses (−1 MU) only in the WL-trials (250 vs. 257 ms, *p < 0.001*); however, during session 2, this difference was insignificant (236 vs. 240, *p = 0.15*). The post hoc comparison also revealed that during session 2, RT in trials with larger and smaller losses significantly differed only in the LL-trials (236 vs. 239 ms, p *< 0.0001*). Overall, during two sessions, the performance of the participants improved on the MID task, and they reacted differently to large and small losses, particularly when it was relevant for maximising monetary outcomes.

Our behavioural data revealed that during two sessions, participants successfully learned to perform the MID task faster, and they also reacted differently to large and small losses, particularly when it was essential for maximising monetary outcomes. However, if the effect of *Day* amounted to 16 ms or a 6% decrease in response time, the effect of *Loss size* was much smaller (about 2 ms), within the range of a keyboard delay. The absence of a clear effect of *Loss size* or of the *Loss size × Day × Loss context* interactions might be explained by a ceiling effect on the RT of a relatively simple MID task, which attenuated the differences between sessions. Furthermore, the adaptive algorithm of the MID task, which keeps the performance around 60%, could also affect RTs since it continuously adjusts task difficulty. Importantly, minute learning-related changes of RT, which were observed in this study, replicate our previous finding of small but significant learning-related changes to RT, evoked by the MID task.

***MMN results***

Although the amplitudes of the MMN responses visually differed across three outcome contexts already in Session 1 (see Figure 2 in the main text of the article), we found no statistical evidence to support significant difference of MMN in LL-trials, HL-trials and WL-trials for Session 1. We performed additional RM-ANOVA with the factor *Sound Type* (eight trial types: six incentive cues and two control sounds) for MMN amplitudes during the Session 1. The analysis revealed no significant differences between trial types: F (7, 196) = 1.5729, p = 0.1454.

***MMN–FRN correlation***

We further checked the specificity of the observed correlation between the dMMN signature of cortical plasticity and the dFRN signature of reinforcement learning signals for the WL-trials in which participants were motivated to discriminate the incentive cues for maximizing monetary outcomes (see Figure 4 in the main text of the article). For this, we additionally correlated the same dMMN in WL-trials with dFRN in LL-trials, where the participants were not motivated to discriminate incentive cues. We found no significant association: r = –0.03, p = 0.87.
